# Assessing Patterns of Human-Wildlife Conflicts and Compensation around a Central Indian Protected Area

**DOI:** 10.1371/journal.pone.0050433

**Published:** 2012-12-05

**Authors:** Krithi K. Karanth, Arjun M. Gopalaswamy, Ruth DeFries, Natasha Ballal

**Affiliations:** 1 Ecology, Evolution, and Environmental Biology, Columbia University, New York, New York, United States of America; 2 Centre for Wildlife Studies, Bangalore, India; 3 Nicholas School of Environment, Duke University, Durham, North Carolina, United States of America; 4 Wildlife Conservation Research Unit, Department of Zoology, University of Oxford, Tubney, United Kingdom; 5 Wildlife Conservation Society-India program, Bangalore, India; Smithsonian’s National Zoological Park, United States of America

## Abstract

Mitigating crop and livestock loss to wildlife and improving compensation distribution are important for conservation efforts in landscapes where people and wildlife co-occur outside protected areas. The lack of rigorously collected spatial data poses a challenge to management efforts to minimize loss and mitigate conflicts. We surveyed 735 households from 347 villages in a 5154 km^2^ area surrounding Kanha Tiger Reserve in India. We modeled self-reported household crop and livestock loss as a function of agricultural, demographic and environmental factors, and mitigation measures. We also modeled self-reported compensation received by households as a function of demographic factors, conflict type, reporting to authorities, and wildlife species involved. Seventy-three percent of households reported crop loss and 33% livestock loss in the previous year, but less than 8% reported human injury or death. Crop loss was associated with greater number of cropping months per year and proximity to the park. Livestock loss was associated with grazing animals inside the park and proximity to the park. Among mitigation measures only use of protective physical structures were associated with reduced livestock loss. Compensation distribution was more likely for tiger related incidents, and households reporting loss and located in the buffer. Average estimated probability of crop loss was 0.93 and livestock loss was 0.60 for surveyed households. Estimated crop and livestock loss and compensation distribution were higher for households located inside the buffer. Our approach modeled conflict data to aid managers in identifying potential conflict hotspots, influential factors, and spatially maps risk probability of crop and livestock loss. This approach could help focus allocation of conservation efforts and funds directed at conflict prevention and mitigation where high densities of people and wildlife co-occur.

## Introduction

Reducing conflict between wildlife and people is considered a top conservation priority, particularly in landscapes where high densities of people and wildlife co-occur [1]–[2]. Increased visibility for conflict incidents may be attributed to actual increase in incidents taking place or just greater reporting by affected local people [3]. Dearth of knowledge about conflict loss and compensation distribution contributes to poor allocation of conservation resources [4]–[5]. Failure to address emerging issues with conflict losses and distribution of compensation may lead to escalation of tensions between people and wildlife, and promote retaliatory actions leading to extirpations of species [6]–[7]. Preventing conflict and improving distribution of compensation are important to fostering co-existence in landscapes that surround protected areas and function as critical buffers for wildlife [8]–[9].

Indian protected areas (PAs) support a huge array of wildlife that are prone to conflict with people. People tolerate some species such as Nilgai *Boselaphus tragocamelus*, Chinkara *Gazzella bennetti* and Blackbuck *Antilope cervicapra* but are less tolerant of other species such as wild pigs *Sus scorfa* and elephants *Elephas maximus* [10]–[11]. Crop loss is more common than livestock loss, human injury and death [12]. Local residents most often directly bear the costs of living alongside wildlife and may have limited ability to cope with losses [12]. Understanding the factors associated with conflict and where they are likely to occur is important for conservation management of conflicts [2], [13].

In this study, we assess and map perceived conflict and compensation distribution to households around Kanha National Park in Central India. Loss refers to probabilities of surveyed households reporting crop raiding or livestock predation. By spatially modeling and mapping perceived conflict and self-reported compensation distribution, we potentially identify household characteristics and practices along with environmental factors that influence conflict and compensation. Data limitations on the timing and locations of actual conflicts and lack of access to detailed records of compensation restrict us to surveys of local people as the primary source of information (see Methods).

In this paper, we examined:

- What agricultural and demographic factors (e.g., land area, total number of crops grown in a year, cropping months in a year, household members, proportion of men), environmental factors (proximity to PA and water, elevation, forest cover), and mitigation measures (e.g. fencing, lighting, physical structures, night guards) are associated with households’ self-reported conflict?
- How does loss vary between crop raiding versus livestock predation?
- Which demographic factors, conflict type, wildlife species, household location and reporting effort are associated with reported compensation received by households?

We expected households in closer proximity to the PA and water, surrounded by forest cover and in lower elevations with greater availability of forage from crops (numbers or types of crops grown in a year by a household and growing season) to be more prone to conflict [4], [14]–[15]. In contrast, we might expect demographic factors (more household members, more men), household characteristics (such as land size or agricultural income) and use of mitigation measures to lower household loss [16]. We also examined reported compensation distribution around Kanha. We expected households reporting conflict incidents, as well as those with more educated members, larger land size, and more livestock to receive better compensation [14]. We also expected compensation distribution to be influenced by species involved, perhaps better for tigers and leopards compared to ungulates and other carnivores [17]–[19].

## Materials and Methods

### Study Sites

**Figure 1:**
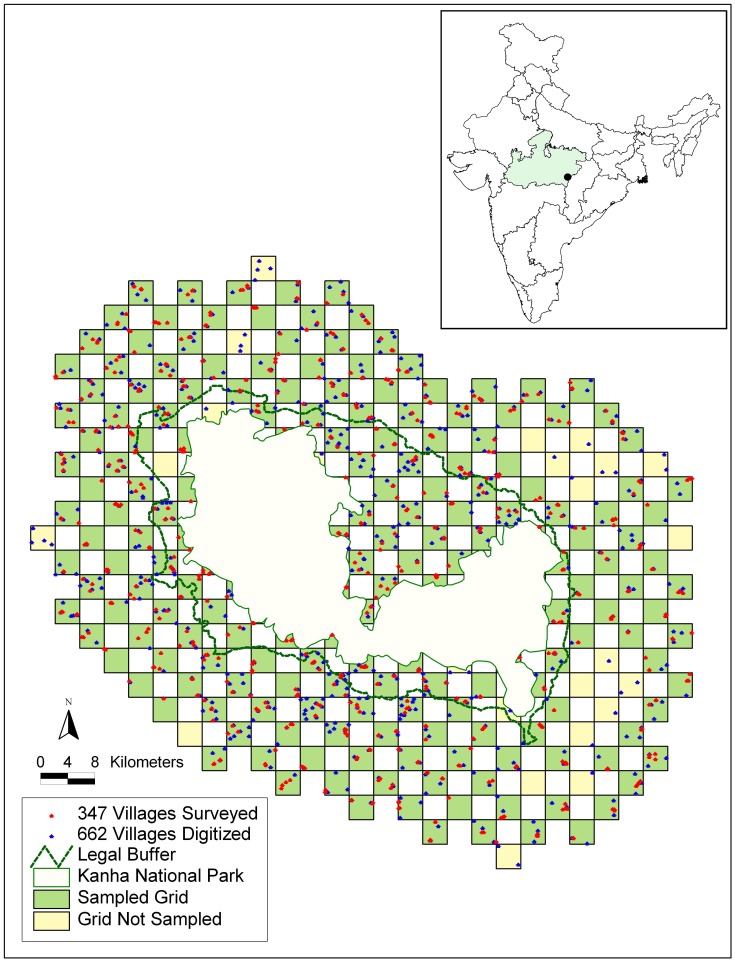
Sampling design and households surveyed around Kanha National Park.

Kanha National Park (22° 7′–22° 27′N, 80° 26′–81° 3′E), established in 1955, is one of India’s most well known Tiger Reserves. Kanha (from here onwards) covers an area of 940 km^2^ and is surrounded by an administrative buffer of 1005 km^2^ (Fig. 1). Vegetation is comprised of Sal and mixed deciduous forests interspersed with grasslands, and supports carnivores such as tiger *Panthera tigris*, leopard *Panthera pardus* and wild dog *Cuon alpinus*, and herbivores including sambar *Cervus unicolor*, chital *Cervus axis*, barasingha *Cervus duvaucelii* and gaur *Bos gaurus* [9]. Local livelihood activities such as grazing and collection of resources are common occurrences in Kanha although all of these activities are legally prohibited inside Kanha [12]. Human population densities range between 182–195/km^2^, and livestock densities range between 65–79/km^2^ in the three districts adjoining the PA [9]. Unlike most other Indian PAs, Kanha has an administratively designated buffer and is surrounded by forested patches that are interspersed with agricultural and barren land [9]. In the administrative buffer activities such as grazing, collection of forest resources and all activities are permitted. The presence of a buffer provides an opportunity to compare conflict experienced and compensation effectiveness among households within and outside the buffer.

### Social Surveys and Interviews

Trained assistants conducted 735 structured and open-ended surveys with households selected from 347 villages surrounding Kanha (Table 1). Although there are households inside the park, we only surveyed households outside the park including within the administrative buffer. To enable systematic spatial sampling, we used a grid based approach to sample 5154 km^2^ area by placing 218 grid cells (13 km^2^ in size) in a 20 km radius outside the PA (Fig. 1). We selected a 20 km radius as a reasonable distance within which we would expect wildlife, particularly ungulates, from the PA to travel outside [20, Karanth et al. unpublished]. Due to the interest in a large geographic area, we sampled 50% of the grid cells in a checkerboard pattern (area of 2319 km^2^), with 193 cells (we excluded 9 cells that were forested without villages, and 16 cells that were logistically inaccessible). Survey of India topographic maps, imagery and Google Earth were used to digitize 632 villages around Kanha (v6.1). Our goal was to sample at least 60% of villages in a cell and survey at least one household from each village. In each village we opportunistically selected and approached individual households. In some cells that had just 1 or 2 villages, we surveyed more than one household in such villages but ensured that these households were located as spatially apart as possible. We were able to opportunistically sample an average of 4 villages in each grid cell (ranging from 1–5 villages in a cell). We approached adult male and female respondents and they were questioned about household demographic and socio-economic characteristics and mitigation measures (use of fencing, lighting, guard animals etc., Table 1). Respondents were specifically questioned about recent (2010 and 2011) experience with all conflict incidents (crop raiding, livestock predation and human injury or death), as well as compensation reported and received. All surveys were conducted in October 2011 in Hindi and recorded responses were translated to English. All survey protocols were approved by Columbia University’s IRB.

**Table 1:** Household characteristics of 735 surveyed households around Kanha.

Characteristics	Sub-characteristics	Household details
Villages Sampled		347
Number of people in HH (average)		6
Livestock* ownership (average/hh)		7
Annual agricultural income (average/hh)		Rs 12070
Distance to park in km (average)		10.1 (0.01–22.2)
Compensation		61% received Rs 1000 to 5000
Top-ranked crop raiding species	a. Wild Pig	48%
	b. Chital	17%
	c. Hanuman Langur	14%
	d. Rhesus Macaque	7%
	e. Jackal	5%
Income loss from crop raiding		Average Rs 4324, Maximum Rs 175000
Top ranked predators	a. Jackal	29%
	b. Wolf	28%
	c. Tiger	22%
	d. Leopard	17%
Income loss from predation		Rs 1078 (maximum Rs 75,000)
HH visited by authorities		18% (67% within a week, 17% within a month and 16% after a month)

We also collected official year wise summary records of total compensation paid out to individual households by the authorities for crop and livestock loss for the years 2009–2011. These records only cover households located inside the PA and administrative buffer. Compensation by the park authorities is determined by the geographic location of households. Households apply to either territorial revenue or forest divisions in which they are located and the authorities compensate those households filing within their administrative division (Karanth et al. unpublished). Typically the amount of compensation provided is determined by officials assessing the extent of damage and do not differentiate among species (Shukla pers comm. 2012) and do not differentiate among species.

### Variable and Model Selection

We used model selection to identify factors associated with self-reported household incidents of crop raiding, livestock predation and compensation distribution around Kanha. The variables chosen are in Table 2. To avoid colinearity of variables used in the model, we computed Pearsons’ correlation coefficients for all pairs of variables. High correlation (>0.5) resulted in elimination of some variables [used distance to PA variable over distance to closest forest patch, (Table S1A–B) [21]–[22]. To permit direct comparison of estimated coefficient values, we scaled the regression inputs by dividing by two standard deviations [23].

**Table 2:** Details on variables collected from surveys and used in models.

Variable	Crop loss	Livestock loss	Compensation
Distance to park	√	√	√
Distance to water	√	√	–
Forest cover	√	√	–
Elevation	√	√	–
Total land area	√	√	√
Gender of respondents	√	√	√
Caste	√	√	√
Total number of people in household	√	√	–
Number of crops	√	–	√
Cropping months	√	–	√
Agriculture land area	√	–	–
Legal agriculture title	√	–	√
Fencing	√	–	–
Night watching	√	√	–
Guard animals	√	–	–
Lighting	√	–	–
Scare devices	√	–	–
Total livestock owned	–	√	√
Grazing goat inside	–	√	–
Grazing cattle inside			
Keeping closer eye on owned animals	–	√	–
Physical structures	–	√	–
Crop loss reported to authorities	–	–	√
Livestock-loss reported to authorities	–	–	√
Human injury reported to authorities	–	–	√
Human death reported to authorities	–	–	√
Household located inside buffer	–	–	√
Any crop raiding mitigation measure	√	–	–
Any livestock predation mitigation measure	–	√	–
Pig related incident	–	–	√
Chital related incident	–	–	√
Langur related incident	–	–	√
Macaque related incident	–	–	√
Jackal related incident	–	–	√
Tiger related incident	–	√	√
Leopard related incident	–	√	√
Jackal related incident	–	√	√
Wolf related incident	–	√	–

Our models represented different hypotheses about factors potentially influencing reported household loss. We defined an a priori set of candidate models, assessed model fit and identified the variables significantly associated with crop and livestock loss using the Corrected Akaike’s Information Criterion (AICc) as the model selection tool since the ratio of the number of data points to the number of structural parameters in our model set was, on average less than 40 [24] (Table S2). Additionally, the AICc criterion is also known to perform well under some degree of correlation among explanatory variables [24]. AICc weights represented the relative measure of appropriateness of a given model relative to the entire model set and rank the models (including a ‘global model’ that includes all explanatory variables of interest). The top model which provided the most optimal trade-off between model parsimony and fit, in our goal to assess association, and closely competing top models (a subset of models accounting for >95% of the model uncertainty) was used to estimate probabilities of crop raiding, livestock predation and compensation distribution by multi-model inference via model averaging [24].

### Modeling Household Risk of Crop-raiding and Livestock Predation

We defined household loss as a household reporting recent experiences with crop raiding or livestock predation (in 2010 and 2011). We chose to ask respondents only about the most recent year as we believed that respondents might forget or confuse incidents over multiple years. Since our responses were binary, we fit logistic regression models to model household conflict loss.

We modeled self-reported household crop raiding and livestock predation as a function of environmental factors. The environmental factors included distance to PA, distance to water, elevation and percentage of forest cover within 3 km of household for each household (data derived based on 9). The selection of 3 km buffer around a household represented a reasonable estimate for home ranges of crop raiding wildlife in tropical forests ([20] and references therein). We also examined the influence of agricultural factors (number of crop types grown in a year, number of cropping months, land area owned by households reported in the surveys). We examined demographic characteristics (number of household members, proportion of men, gender of respondent). We also compared household use of different mitigation efforts: lighting versus fencing versus guard animals. We modeled reported compensation received by households as a function of individual respondents factors (gender, age), household characteristics (land size, total livestock owned), conflict type (crop loss, livestock loss, human injury, human death), reporting effort by households to authorities, wildlife species, proximity to the PA and location inside administrative buffer.

We constructed 31 a priori models representing our hypotheses about causes of ‘crop-raiding’ (Table S2), including a global model with 18 explanatory variables. We modeled household livestock predation as a function of 20 variables represented by 30 models (Table S2). Similarly, we proposed 36 models of compensation distribution using 22 variables (Table S3). We computed the weighted frequency of each variable among all the models to assess the relative importance of each variable. For each surveyed household, we estimated crop loss, livestock loss and compensation access probabilities for all the candidate models. The weighted average of these individual model estimates, weighted by the Akaike weights [24], was used to generate overall estimates for individual households crop loss, livestock loss and compensation access.

### Spatial Mapping of Conflict Risk and Compensation Distribution

We derived estimated probabilities for crop loss, livestock loss and compensation access for every household from each individual model from the entire model set and these values are then weighted to derive model-averaged estimates for each household. We used ordinary kriging to estimate crop loss, livestock loss and compensation distribution probabilities in non-sampled areas. Kriging is a geostatistical interpolation technique that permits inferences about a parameter of interest (in our case, the probability of conflict or compensation) in non-sampled areas by using information available in sampled locations and accounting for uncertainty as the distance between spatial locations increases. The approach produces a semivariogram that describes the spatial correlation between the points. Several models are available for fitting a semivariogram, such as spherical, circular, exponential, Gaussian and linear [25]–[26]. Based on the Akaike’s information criterion (AIC), we fit the spherical model to the observed data, using the Kriging Interpolator 3.2 extension in ArcView Spatial Analyst [27]. In our application, we treated the weighted estimate of conflict loss probability at each sampled location as “truth”, assuming no variability around the estimate (i.e. no nugget effect) [28], to generate maps of conflict loss probabilities for crop loss and livestock predation, as well as distribution of compensation reported by households around Kanha.

## Results

### 1 Household Characteristics, Agricultural Practices and Reported Crop Loss

Household respondents were largely men (87%), with many (77%) having completed 8^th^ grade or less education. Majority of households (93%) were engaged in agriculture, 88% legally owned land with average land size of 4.3 acres. Households reported growing 12 different crops. The major crops grown were rice, millet and legumes, and the average length of time for crops in the field was 5 months (range 4–12 months, Table 1).

**Figure 2:**
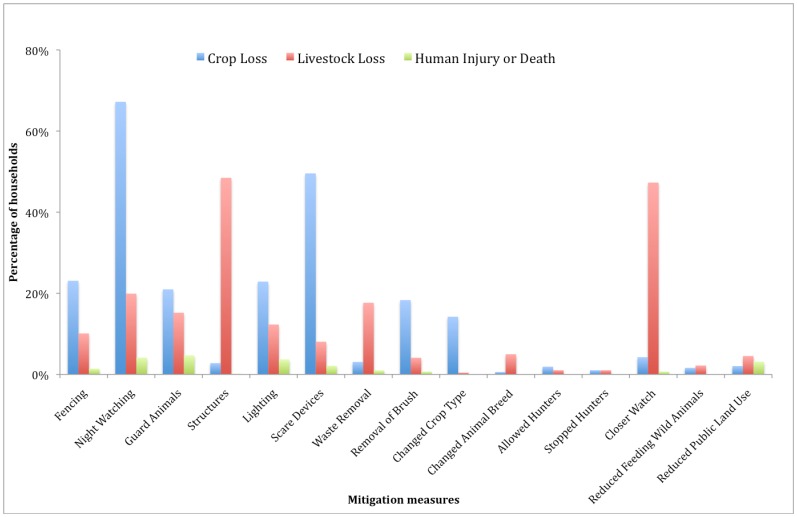
Mitigation measures reported by surveyed households around Kanha National Park.

Households reporting crop loss listed 17 species as crop raiders including 10 herbivores, 4 carnivores, 2 primates and peacocks. Animals reported causing the most crop damage were wild pig *Sus scrofa*, chital and langur *Semnopithecus entellus* and used multiple mitigation measures (Table 1, Fig. 2). The highest numbers of raiding incidents were reported from September to December, peaking in October. Sixty-four percent of households reported experiencing more than five incidents per year and 32% households reported 2–5 incidents per year.

From modeling factors associated with self-reported household crop loss, we found five top-ranked models with substantial weights (cumulative AICc >0.95, Table 3). As predicted, the total number of crops grown in a year was associated with increased households’ loss (positive *β* coefficient in Table 3) but no other household characteristics were relevant. Although use of mitigation measures, in general, appeared important, no individual measure, such as fencing or lighting, appeared noticeably more important (Table 3). We suspect that the strong positive associations of any general mitigation measure (as indicated by positive beta coefficients in Table 3) are a result of these mitigation measures being put in place because households have historically experienced many incidents of crop raiding. Among the environmental factors distance to PA was associated with decreased crop loss (negative beta coefficient in Table 3). Distance to water was associated with increased household loss (positive beta coefficient in Table 4) but high uncertainty in the estimates suggested that this parameter must be interpreted with caution. Contrary to our expectations other modeled factors did not appear to influence crop loss (Table 2 and Table S2), although we recognize that a much larger sample size might have assisted in providing greater support for other explanatory factors as well.

**Table 3:** Top models (cumulative weight>0.95) and beta coefficients for predicting household crop loss around Kanha National Park.

Models	30	29	27	26	28
	w_i_ = 0.35	w_i_ = 0.28	w_i_ = 0.17	w_i_ = 0.12	w_i_ = 0.07
Intercept	1.62 (0.11)	1.62 (0.11)	1.62 (0.11)	1.63 (0.11)	1.60 (0.11)
Number of crops	1.02 (0.25)	1.03 (0.25)	1.02 (0.25)	0.94 (0.25)	0.89 (0.23)
Average cropping months	−0.37 (0.21)	−0.40 (0.21)	−0.40 (0.21)	−0.53 (0.22)	NA
Agriculture land area	NA	NA	NA	−0.06 (0.20)	NA
Legal agriculture title	NA	NA	NA	0.51 (0.27)	NA
Use of any mitigation measure	1.84 (0.22)	1.88 (0.22)	1.86 (0.22)	1.82 (0.22)	1.77 (0.21)
Distance to PA	−0.55 (0.21)	−0.55 (0.21)	−0.54 (0.21)	−0.50 (0.21)	−0.56 (0.21)
Distance to water	NA	NA	0.22 (0.21)	0.21 (0.21)	0.22 (0.21)
Forest cover	NA	−0.25 (0.20)	−0.27 (0.20)	−0.30 (0.20)	−0.23 (0.20)
Model AICc	636.67	637.14	638.08	638.8	639.75
Δ AICc	0	0.47	1.41	2.13	3.08

* Note: Standard errors in brackets and top-ranked models are shown, wi is the AIC model weight **Δ**AICc is the difference in values between lowest AIC model and each model.

**Table 4:** Top models (cumulative weight >0.95) and beta coefficients for predicting household livestock loss around Kanha National Park.

Models	27	28	5	3	29	4	6	2	1
	w_i_ = 0.25	w_i_ = 0.19	w_i_ = 0.16	w_i_ = 0.12	w_i_ = 0.09	w_i_ = 0.08	w_i_ = 0.06	w_i_ = 0.02	w_i_ = 0.02
Intercept	0.24 (0.08)	0.24 (0.08)	0.24 (0.08)	0.24 (0.08)	0.24 (0.08)	0.24 (0.08)	0.24 (0.08)	0.24 (0.08)	0.24 (0.08)
Distance to PA	−0.93 (0.16)	−0.93 (0.16)	−0.97 (0.16)	−0.94 (0.16)	−0.93 (0.16)	−0.95 (0.16)	−0.97 (0.16)	−0.83 (0.17)	−0.95 (0.16)
Distance to water	0.46 (0.16)	0.47 (0.16)	0.49 (0.16)	0.46 (0.16)	0.46 (0.16)	0.47 (0.16)	0.49 (0.16)	0.40 (0.18)	0.51 (0.16)
Forest cover	−0.05 (0.16)	−0.03 (0.16)	−0.05 (0.16)	−0.06 (0.16)	−0.05 (0.16)	−0.05 (0.16)	−0.05 (0.16)	−0.03 (0.15)	−0.04 (0.15)
Elevation	NA	NA	NA	NA	NA	NA	NA	0.29 (0.19)	NA
Grazing cows inside PA	0.42 (0.19)	0.40 (0.19)	0.41 (0.19)	0.43 (0.19)	0.42 (0.19)	0.42 (0.19)	0.41 (0.19)	NA	NA
Night watching	NA	NA	NA	0.38 (0.21)	NA	0.37 (0.20)	NA	NA	NA
Physical structures	NA	NA	NA	−0.08 (0.16)	NA	−0.08 (0.16)	NA	NA	NA
Any mitigation measure	0.29 (0.17)	0.30 (0.17)	NA	NA	0.29 (0.17)	NA	NA	NA	NA
Total number of people in household	NA	0.19 (0.16)	NA	NA	NA	0.16 (0.16)	NA	NA	NA
Total livestock	NA	NA	NA	NA	0.001 (0.16)	NA	0.04 (0.16)	0.09 (0.16)	0.10 (0.16)
Model AICc	961.53	962.12	962.55	963.07	963.57	964.03	964.53	966.74	967.03
Δ AICc	0	0.59	1.03	1.54	2.04	2.5	3	5.21	5.5

¶ Note: Standard errors in brackets and top-ranked models are shown, wi is the AIC model weight **Δ** AICc is the difference in values between lowest AIC model and each model.

**Figure 3:**
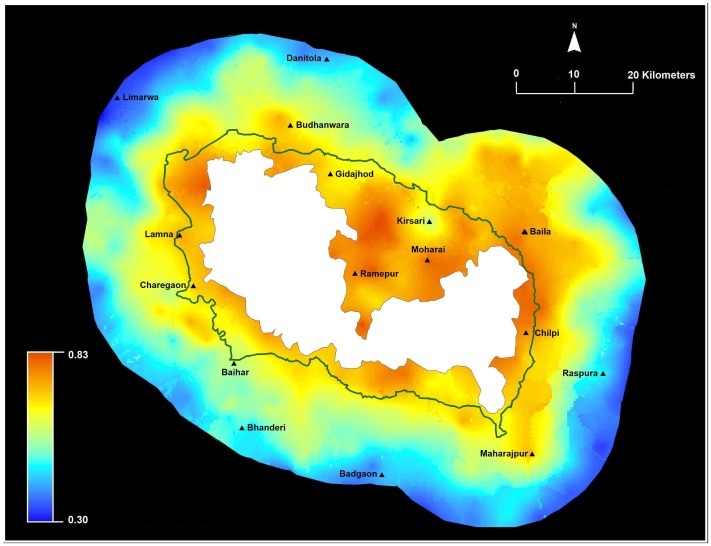
Predicted crop loss within 20 km around Kanha National Park (dark green is the administrative buffer). Kriging generates probabilities for crop loss in the landscape with blue areas depicting low risk and red areas depicting high risk.

We mapped the probability of crop loss for households around Kanha (Fig. 3). Our modeling indicated that crop loss risk was high in most places, average estimated probabilities of crop loss was 0.93 (range 0.66–0.99). Probabilities of crop loss averaged for households inside the administrative buffer was 0.95 (S.E = 0.004, range 0.77–0.99) and for households outside the buffer was 0.92 (S.E = 0.003, range 0.66–0.99). Spatial modeling based on ordinary kriging (without the nugget effect) suggests that households located closer to the PA have higher risk regardless of location within or outside the administrative buffer. The spatial model locates hot spots of conflict (Fig. 3) and identified high risk particularly around some villages in the buffer (Ramepur, Moharai, Kirsari), and for other villages outside the buffer (Baila, Budhanwara, Maharajpur, Khorja).

### 2 Livestock Ownership and Perceived Predation Risk

Most (91%) of households owned livestock and ninety percent of animals were stall fed or locally grazed compared to 10% being park grazed. Noticeably households reported an increase in stall-feeding by 7.6% over 10 years and an increase in the number of days their animals were grazed in the last ten years (Table 1). This is perhaps indicative of declining forage areas in close proximity to people’s households requiring them to graze livestock further away from home and well inside the PA.

Livestock losses to ten carnivores were reported and most troublesome species were jackal *Canis aureus*, wolf *Canis lupus*, tiger and leopard (Table 1). Livestock losses were distributed across the year but higher from April through July, which are the driest summer months in Kanha (9). Some (34%) households reported more than five incidents per year, 34% households reported 2–5 incidents per year and 31% reported one incident per year. Seven-percent of households reported experiencing injury and 1% reported instances of human death (Table 1).

From modeling factors associate with self-reported household risk to livestock loss outside Kanha, we identified nine top-ranked models (cumulative AICc >0.95, Table 4). These models indicate that as predicted grazing cows inside Kanha was associated with increased loss (positive beta coefficient in Table 4). Among the environmental factors, distance to PA was associated with decreased livestock loss (negative beta coefficient in Table 4). Distance to water was associated with increased household loss (positive beta coefficient in Table 4) but high standard errors suggest caution with interpreting this result. Two mitigation factors night watching and physical structures appeared in top ranked models (Table 3). Only use of physical structures (negative beta coefficient in Table 4) was associated with lower losses but high standard errors suggest that this must be interpreted with caution. Other modeled factors did not appear to be associated with household livestock loss (Table S2).

**Figure 4:**
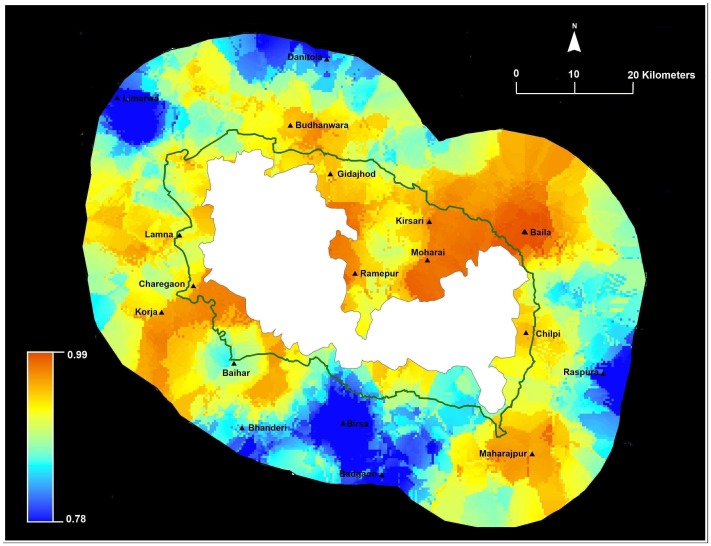
Predicted livestock predation loss within 15 km around Kanha National Park (dark green is the administrative buffer). Kriging generates probabilities for livestock loss in the landscape with blue areas depicting low risk and red areas depicting high risk.

We mapped risk of livestock predation for households around Kanha (Fig. 4). Our modeling clearly indicates that livestock loss risk occurs in some places and average estimated probability was 0.60 (range 0.30–0.85). Probability of livestock loss averaged for households inside the administrative buffer was 0.74 (S.E = 0.004, range 0.57–0.85) and loss probability averaged for households outside buffer was 0.55 (S.E = 0.005, range 0.30–0.83). Spatial modeling by ordinary kriging identified hot spots of risk around the villages of Ramepur, Moharai and Chilpi inside the buffer and Baila outside the buffer (Fig. 4).

### 3 Mitigation Measures

Households reported several mitigation measures used by them to protect property and lives. To protect crops the most common mitigation measures were night watching (67%) and scare devices (49%) and to protect livestock measures deployed by households were closer watch on animals (48%) and physical structures (47%, Table 1, Fig. 2). Our models suggest that potentially physical structures and guard animals appeared to be associated with lower crop loss, and no mitigation appeared to be associated lower livestock loss.

### 4 Compensation Distribution

Although 73% of those surveyed reported that they experienced crop loss, only 26% of these households reported losses to authorities and 22% of those reporting received compensation (ranging from $21 to $104, 1 US$ = 48 Indian Rupees at the time of the study). Most (63%) households reported losing up to 75% of crops to wildlife (Table 1). Authorities reportedly visited 18% of affected households, and 68% of visits were within the first week. In Kanha the average household reported annual loss of income from crop loss was US $90 (where average annual income is $517 per household).

Thirty percent of surveyed households reported to have experienced livestock loss, but only 34% of these affected households reported losses to authorities and 41% received compensation upon reporting. In Kanha official compensation paid from 2006–2011 to 524 households inside the PA was $84 and higher than the $63 compensation paid to 1644 households located in the buffer (Kanha Forest Department 2012). In our study, households reported receiving compensation averaging $22 (ranging $20–100). Although we cannot directly compare official compensation paid to individual households, the financial amounts of compensation from our surveyed households compare with official records. Surveys conducted in parks around Karnataka also find similar self-reporting levels of loss and compensation ([12], Karanth et al. unpublished).

From modeling factors associated with reported household access to compensation we identify important factors associated with compensation receipt (Table 5). Households filing claims to authorities and located in the administrative buffer were more likely to receive compensation and this is supported by positive beta coefficients for reporting crop and livestock loss (Table 5). Species involved was also associated with compensation received by households, particularly reporting tigers had positive influence (positive beta coefficient in Table 5). Conflict incidents related to other species do not appear to have yielded compensatory benefits to households (negative beta coefficients in Table 5). Reporting incidents of human injury and death occur in fewer models but did not appear to be associated with improved compensation (positive beta coefficients in Table 5). Households’ location in the legal buffer appeared to be associated with higher compensation (positive beta coefficients in Table 5). Other modeled factors did not appear to influence compensation (Table S3).

**Table 5:** Top models (cumulative weight >0.95) and beta coefficients for predicting household compensation distribution around Kanha National Park.

Models	26	27	28	30	29	11	31	6
	w_i_ = 0.37	w_i_ = 0.31	w_i_ = 0.10	w_i_ = 0.08	w_i_ = 0.06	w_i_ = 0.04	w_i_ = 0.03	w_i_ = 0.01
**Intercept**	−2.93 (0.19)	−2.99 (0.20)	−2.95 (0.19)	−3.00 (0.20)	−2.93 (0.19)	−2.87 (0.18)	−2.97 (0.20)	−2.93 (0.19)
**Crop-raiding reported to authorities**	0.52 (0.38)	0.58 (0.38)	0.58 (0.38)	0.50 (0.38)	0.48 (0.39)	0.66 (0.37)	0.50 (0.39)	0.74 (0.37)
**Livestock-predation reported to authorities**	1.44 (0.44)	1.49 (0.45)	1.53 (0.45)	1.47 (0.46)	1.42 (0.44)	1.63 (0.43)	1.51 (0.46)	1.67 (0.45)
**Wild pig**	−0.83 (0.41)	−0.79 (0.41)	−0.74 (0.41)	−0.76 (0.42)	−0.83 (0.41)	−0.75 (0.40)	−0.71 (0.42)	−0.69 (0.40)
**Chital**	−0.05 (0.34)	−0.14 (0.35)	−0.08 (0.35)	−0.11 (0.35)	−0.03 (0.34)	−0.07 (0.34)	−0.05 (0.35)	−0.14 (0.35)
**Tiger**	1.42 (0.40)	1.44 (0.41)	1.37 (0.41)	1.4 (0.42)	1.40 (0.40)	1.49 (0.41)	1.33 (0.42)	1.50 (0.42)
**Leopard**	−0.08 (0.40)	0.05 (0.41)	0.09 (0.41)	0.05 (0.42)	−0.07 (0.4)	−0.13 (0.39)	0.10 (0.42)	−0.002 (0.41)
**Langur**	NA	−0.61 (0.43)	−0.36 (0.40)	−0.74 (0.45)	NA	NA	−0.51 (0.43)	−0.65 (0.43)
**Wolf**	NA	−0.42 (0.48)	−0.46 (0.48)	−0.51 (0.50)	NA	NA	−0.55 (0.50)	−0.47 (0.48)
**Jackal**	NA	−0.28 (0.45)	−0.19 (0.45)	−0.31 (0.46)	NA	NA	−0.23 (0.46)	−0.12 (0.44)
**Bonnet macaque**	NA	1.00 (0.47)	NA	0.98 (0.48)	NA	NA	NA	0.78 (0.46)
**Human injury reported to authorities**	NA	NA	NA	0.66 (0.56)	0.41 (0.53)	NA	0.68 (0.57)	NA
**Human death reported to authorities**	NA	NA	NA	0.24 (0.89)	−0.19 (0.85)	NA	0.37 (0.90)	NA
**Household located inside buffer**	0.83 (0.32)	0.95 (0.33)	0.84 (0.32)	0.96 (0.33)	0.84 (0.32)	NA	0.86 (0.32)	NA
**Model AIC**	346.33	346.69	348.91	349.34	349.85	350.88	351.23	352.79
**Δ AIC**	0	0.36	2.58	3.01	3.52	4.54	4.89	6.46

† Note: Standard errors in brackets and top-ranked models are shown, wi is the AIC model weight ΔAICc is the difference in values between lowest AIC model and each model.

We compared losses experienced and reported to authorities, and compensation received by households inside and outside the buffer. Households reporting crop loss was similar inside and outside the buffer but households reporting livestock loss was higher inside the buffer (Table 6). We find that households inside the administrative buffer were more likely to report losses (46% for crop loss and 62% for livestock loss) compared to households outside the buffer (19% for crop loss and 18% for livestock loss). Of the households that reported loss to authorities, households inside the buffer were more likely to receive compensation (35% for crop loss and 48% for livestock loss) compared to households outside (11% for crop loss and 29% for livestock loss).

**Table 6:** Comparison loss and compensation reported by households surveyed inside and outside administrative buffer around Kanha National Park.

Features	Inside Buffer	Outside Buffer	Total
Number of Households	190	543	733
HH experiencing crop loss	145 (76%)	391 (72%)	536 (73%)
HH reporting crop loss to authorities	66 (46%)	73 (19%)	139 (26%)
HH reporting crop loss to authorities receiving compensation	23 (35%)	8 (11%)	31 (22%)
HH experiencing livestock loss	87 (46%)	153 (28%)	240 (33%)
HH reporting livestock loss to authorities	54 (62%)	28 (18%)	82 (34%)
HH reporting loss to authorities receiving compensation	26 (48%)	8 (29%)	34 (41%)
Survey estimated probability of crop loss	0.95 (SE = 0.004)	0.92 (SE = 0.003)	0.93 (SE = 0.003)
	Range: 0.77–0.99	Range: 0.66–0.99	Range 0.66–0.99
Survey estimated probability of livestock loss	0.74 (SE = 0.004)	0.55 (SE = 0.005)	0.60 (SE = 0.005)
	Range: 0.57–0.85	Range: 0.30–0.83	Range 0.30–0.85
Survey estimated probability of compensation distribution	0.21 (SE = 0.02)	0.05 (SE = 0.003)	0.09 (SE = 0.005)
	Range: 0.03–0.72	Range: 0.01–0.45	Range 0.01–0.72

**Figure 5:**
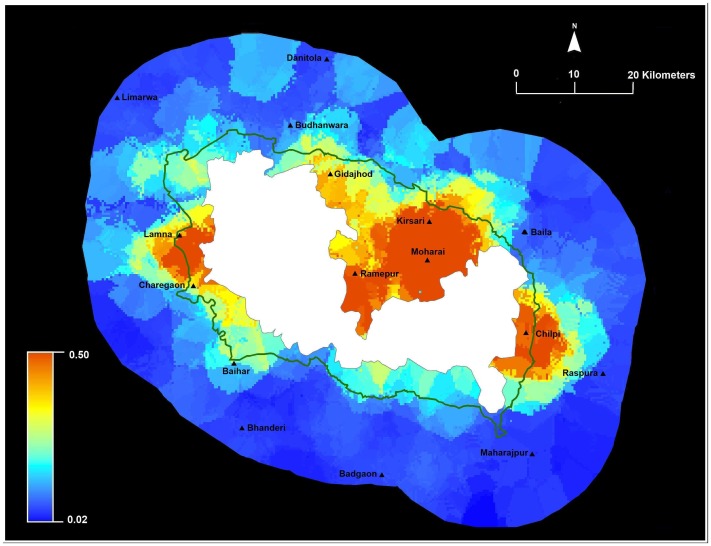
Predicted compensation distribution for households within 20 km around Kanha National Park. Household probabilities of receiving compensation in the landscape are generated by Kriging with blue areas depict low compensation and red areas depicting high compensation.

We mapped reported compensation distribution for households around Kanha. The estimated probabilities averaged 0.09 (range 0.01–0.72, Fig. 5). Households located within the administrative buffer averaged 0.21 (S. E = 0.02, range 0.03–0.72) compared to households outside 0.05 (S. E = 0.003, range 0.01–0.45). Compensation distribution for households located in the administrative buffer is higher than households located outside indicating some positive influence of management inside the buffer. Our spatial model suggests higher compensation around Moharai, Ramepur, Kirsari, Lamna, Umaerdehi and Chilpi inside the buffer (Fig. 5).

## Discussion

Our results identified and quantified main factors associated with self-reported household crop raiding and livestock predation losses, as well as compensation distribution (Figures 3, 4, and 5), in 5145 km^2^ area surrounding Kanha. Similar to other studies, we find spatial and temporal variation in conflict losses [4], [14], [18]. Overall, the probability of crop loss was high (average 0.93) for households and higher for households located within the administrative buffer. Crop loss was associated with greater cropping months and proximity to Kanha. No individual mitigation measure was associated with lowering crop loss. Overall, the probability of livestock loss was comparatively lower compared to crop loss (average 0.60) and higher for households located in the administrative buffer compared to households located outside. Livestock loss was associated with households grazing cows inside Kanha and located closer to the reserve. However, use of physical structures to protect livestock appeared to be the only mitigation measure associated with lowering of livestock loss. Interestingly, over the last ten years households reported a gradual increase in stall-feeding practices compared to free range grazing, suggesting the gradual modification of behavior. This result, however, may merely be a reflection of the inability of people to recall practices from the past. Our findings mirror other studies, where crop and livestock loss are a function of multiple factors, with individual choices and behavior influencing loss and often difficult to modify [29].

Considerable conservation attention and monies are currently being invested in mitigation factors that have rarely been evaluated for effectiveness [16], [30]–[31]. We find only use of physical structures was potentially associated with lowering livestock loss. Our results indicate that blind investment in mitigation activities should be replaced by targeted and focused strategies that work at the individual household level.

Although 73% of households reported crop loss and 33% reported livestock loss, only 26% reported crop loss and 34% reported livestock loss to authorities. Among reporting households, 22% received compensation for crop loss and 41% received compensation for livestock loss.

Likelihood of compensation distribution received by households was higher for households that report incidents related to tigers (although most damage was reported by pig, jackal and wolf incidents, Table 1) and households located in the buffer. There is considerable debate about people’s ability to accurately report losses to authorities, and other studies have found mismatches to official records [2], [14], [32]. These reported losses determined by surveying people have to be complemented with field-based monitoring of households over time. We do find among surveyed households in the administrative buffer were twice as likely to report crop loss and thrice as likely to report livestock loss compared to outside (Table 6). Households in the administrative buffer were more likely to receive compensation from authorities, suggesting that the legal designation and management of an administrative buffer improves reporting of losses and receipt of compensation by households (Table 6).

Overall, many (87%) households around Kanha report experiencing some kind of conflict incident with wildlife and this is comparatively higher than other places in the world [2], [33]. However, there appears to be some degree of acceptance among people that there will be some losses to wildlife (Karanth pers. obs 2011, Karanth et al. unpublished), and perhaps households have evolved coping strategies [11]–[12], [17], [34].

Unlike most Indian PAs, Kanha has a legal administratively designated management buffer [9]. We estimated higher risk of crop and livestock loss inside the buffer, and find higher compensation in the buffer. This corroborates with other studies that suggest delineation of buffers or other management strategies should be based on ecological and economic realities [9]. Additionally, in this landscape we found household distance to the PA was more relevant for livestock and crop loss compared to distance to water. Our results potentially suggest that some crop losses to wildlife from the PA but we know that wildlife naturally persists outside the PA and have overlapping resource use with people and domestic animals (forage and water, Karanth et al. unpublished). So this raises important questions about who is responsible for compensating local people for wildlife damage outside the jurisdiction of PAs and park authorities [6], [35]. Our spatial risk maps (Figs. 3 and 4) clearly identified locations that are hotspots for crop and livestock loss and can guide managers in improving existing conflict prevention and mitigation practices.

### Management Implications

Most conflict studies are characterized by poor spatial sampling and modeling [36]–[37]. To improve efficacy and efficiency of conservation actions, managers require surveying and modeling approaches that are spatially explicit and rigorous [5], [7]. Our approach identified factors associated with crop and livestock loss around Kanha National Park in India. We were able to link ground based survey data to landscape level data and spatially map conflict crop and livestock loss, as well as compensation distribution to identify conflict hotspots and focus ground based conservation efforts. For example, villages and households located in high-risk areas can be better educated about how to prevent and mitigate conflict and can report losses to authorities in an organized manner.

A monitoring system that systematically records and disperses information on conflict is much needed. Such an approach can guide the development of a risk database, and develop live warning and monitoring systems (such as those cell phone alert systems currently in use for Elephants in Valparai in southern India). Experiences from Uganda, Kenya and Sumatra suggest that establishing and long-term maintenance of monitoring systems in local communities remains a considerable challenge and is often difficult to sustain [15], [30], [38]. However, greater participation and involvement of multiple stakeholders (local households, PA authorities) can considerably decrease hostility towards wildlife species [29], [35], [39].

Kanha is atypical of most Indian PAs because the administrative buffer gives it a lesser hard edge than other PAs and livelihoods of people living in the buffer fall within the purview of park management [9]. As consideration is currently under way for designating buffers around other protected areas in India, our results from Kanha suggest that compensation is more likely to be distributed to those who suffer losses if the administrative buffer is designated to include more susceptible locations for crop and livestock loss. This conclusion may apply to other parks located in human-dominated landscapes, though cultural and ecological factors will vary.

Our study has some limitations. The analysis relied primarily on individuals’ recall and self-reporting obtained through surveys and did not observationally monitor actual incidents of conflict. This raises possibilities for over-reporting or exaggeration of losses, although there are no indications of a systematic bias across so many households. The number of surveys is large relative to most published studies and field-based human-wildlife conflict surveys that reduced the potential biases (although we recognize the limitations of self-reported data). To complement the surveys and provide objective data on human wildlife conflicts, active location based monitoring of households to measure conflict as it happens along with assessment of mitigation measures and compensation distribution process is much needed. Extension of surveys across multiple years is also needed to assess additional factors that might influence conflict risk that may not be identified in a single year assessment. Overall, our approach for identifying associated factors and spatially mapping risk is easily extendable to other landscapes where people and wildlife co-occur, so that allocation of physical efforts and funds are more effectively targeted at conflict prevention and mitigation.

## Supporting Information

Table S1 **Correlation matrices for variable selection for crop loss and livestock loss.**
(DOC)

Table S2 **Models included in the model sets for crop and livestock loss.**
(DOC)

Table S3 **Models included in the model set for compensation distribution.**
(DOC)
