# Supplementary material for: Assessing Patterns of Human-Wildlife Conflicts and Compensation around a Central Indian Protected Area
Source: PLoS One. 2012 Dec 5;7(12):e50433. doi: 10.1371/journal.pone.0050433 (PMC3515612; doi:10.1371/journal.pone.0050433)
Supplement: Table S1 — Correlation matrices for variable selection for crop loss and livestock loss. (DOC) [file pone.0050433.s001.doc]

**Table S1a**

|  | *d* | *dw* | *fc* | *e* | *land* | *g* | *caste* | *hht* | *ncrop* | *crmn* | *agramt* | *agrtit* | *ma* | *mb* | *mc* | *me* | *mf* | *cmgen* |
| --- | --- | --- | --- | --- | --- | --- | --- | --- | --- | --- | --- | --- | --- | --- | --- | --- | --- | --- |
| d | 1 |  |  |  |  |  |  |  |  |  |  |  |  |  |  |  |  |  |
| dw | -0.02 | 1 |  |  |  |  |  |  |  |  |  |  |  |  |  |  |  |  |
| fc | 0.00 | 0.13 | 1 |  |  |  |  |  |  |  |  |  |  |  |  |  |  |  |
| e | -0.41 | 0.41 | 0.04 | 1 |  |  |  |  |  |  |  |  |  |  |  |  |  |  |
| land | -0.03 | 0.02 | 0.03 | 0.04 | 1 |  |  |  |  |  |  |  |  |  |  |  |  |  |
| g | 0.00 | 0.02 | 0.08 | -0.02 | 0.00 | 1 |  |  |  |  |  |  |  |  |  |  |  |  |
| caste | -0.04 | 0.10 | -0.04 | 0.25 | -0.07 | -0.04 | 1 |  |  |  |  |  |  |  |  |  |  |  |
| hht | 0.01 | -0.05 | -0.06 | 0.00 | 0.17 | 0.02 | 0.01 | 1 |  |  |  |  |  |  |  |  |  |  |
| ncrop | 0.01 | -0.05 | -0.06 | 0.00 | 0.17 | 0.02 | 0.01 | 1 | 1 |  |  |  |  |  |  |  |  |  |
| crmn | 0.09 | -0.02 | -0.06 | -0.01 | 0.05 | 0.03 | 0.08 | -0.02 | -0.02 | 1 |  |  |  |  |  |  |  |  |
| agramt | 0.03 | -0.06 | -0.03 | -0.07 | 0.41 | 0.04 | -0.08 | 0.12 | 0.12 | 0.04 | 1 |  |  |  |  |  |  |  |
| agrtit | -0.08 | 0.04 | 0.04 | 0.11 | 0.12 | 0.05 | 0.06 | 0.06 | 0.06 | 0.27 | 0.09 | 1 |  |  |  |  |  |  |
| ma | 0.05 | 0.08 | 0.08 | 0.01 | -0.01 | -0.05 | 0.06 | -0.02 | -0.02 | 0.08 | 0.10 | 0.03 | 1 |  |  |  |  |  |
| mb | -0.11 | 0.06 | 0.08 | 0.10 | 0.05 | 0.01 | 0.06 | -0.03 | -0.03 | 0.12 | -0.04 | 0.18 | 0.09 | 1 |  |  |  |  |
| mc | 0.08 | 0.06 | 0.08 | 0.00 | 0.02 | -0.06 | 0.02 | -0.03 | -0.03 | 0.08 | 0.03 | 0.10 | 0.23 | 0.10 | 1 |  |  |  |
| me | 0.02 | 0.01 | 0.07 | -0.02 | -0.02 | 0.07 | 0.00 | -0.07 | -0.07 | 0.13 | 0.02 | 0.04 | 0.10 | 0.28 | 0.22 | 1 |  |  |
| mf | -0.08 | 0.07 | 0.02 | 0.03 | 0.10 | 0.06 | -0.02 | -0.07 | -0.07 | 0.10 | 0.08 | 0.12 | 0.09 | 0.40 | 0.12 | 0.33 | 1 |  |
| cmgen | -0.05 | 0.07 | 0.07 | 0.09 | 0.05 | 0.01 | 0.06 | -0.05 | -0.05 | 0.20 | 0.05 | 0.26 | 0.28 | 0.69 | 0.27 | 0.28 | 0.50 | 1 |

Note: d=distance to Kanha national park, dw = distance to water, e= elevation, fc=forest cover within 3km of each household, g= gender(male,female), caste= caste (upper caste, other backward classes, scheduled caste, scheduled tribe), hht = total number of people in household, land = total land area, ncrop = number of crops, crmn=average number of cropping months, agramt= agriculture land area, agrtit= legal agriculture title, ma= fencing, mb = night watching, mc=guard animals, md = physical structures , me =lighting, mf =scare devices, cmgen=any mitigation measure used for crop-raiding

**Table S1b**

|  | *d* | *dw* | *fc* | *e* | *totlvs* | *g* | *caste* | *hht* | *land* | *grpab* | *grpag* | *grpac* | *ltgr* | *llpd* | *ljkl* | *lwlf* | *mb* | *mm* | *md* | *lmgen* |
| --- | --- | --- | --- | --- | --- | --- | --- | --- | --- | --- | --- | --- | --- | --- | --- | --- | --- | --- | --- | --- |
| d | 1 |  |  |  |  |  |  |  |  |  |  |  |  |  |  |  |  |  |  |  |
| dw | -0.02 | 1 |  |  |  |  |  |  |  |  |  |  |  |  |  |  |  |  |  |  |
| fc | 0.00 | 0.13 | 1 |  |  |  |  |  |  |  |  |  |  |  |  |  |  |  |  |  |
| e | -0.41 | 0.41 | 0.04 | 1 |  |  |  |  |  |  |  |  |  |  |  |  |  |  |  |  |
| totlvs | -0.01 | -0.01 | 0.02 | 0.02 | 1 |  |  |  |  |  |  |  |  |  |  |  |  |  |  |  |
| g | 0.00 | 0.02 | 0.08 | -0.02 | 0.05 | 1 |  |  |  |  |  |  |  |  |  |  |  |  |  |  |
| caste | -0.04 | 0.10 | -0.04 | 0.25 | -0.05 | -0.04 | 1 |  |  |  |  |  |  |  |  |  |  |  |  |  |
| hht | 0.01 | -0.05 | -0.06 | 0.00 | 0.16 | 0.02 | 0.01 | 1 |  |  |  |  |  |  |  |  |  |  |  |  |
| land | -0.03 | 0.02 | 0.03 | 0.04 | 0.10 | 0.00 | -0.07 | 0.17 | 1 |  |  |  |  |  |  |  |  |  |  |  |
| grpab | 0.04 | 0.12 | 0.01 | 0.09 | 0.22 | 0.03 | -0.02 | 0.01 | -0.03 | 1 |  |  |  |  |  |  |  |  |  |  |
| grpag | -0.06 | 0.02 | 0.01 | 0.06 | 0.33 | 0.06 | -0.01 | 0.04 | 0.01 | 0.22 | 1 |  |  |  |  |  |  |  |  |  |
| grpac | 0.05 | 0.10 | 0.04 | 0.10 | 0.18 | 0.02 | 0.06 | 0.05 | -0.08 | 0.33 | 0.26 | 1 |  |  |  |  |  |  |  |  |
| ltgr | -0.21 | 0.10 | -0.04 | 0.15 | 0.11 | 0.02 | 0.02 | 0.00 | 0.04 | 0.02 | -0.01 | 0.00 | 1 |  |  |  |  |  |  |  |
| llpd | -0.12 | 0.18 | -0.01 | 0.20 | 0.05 | 0.02 | 0.09 | -0.03 | 0.02 | 0.05 | 0.00 | 0.06 | 0.45 | 1 |  |  |  |  |  |  |
| ljkl | -0.09 | 0.00 | -0.03 | 0.15 | 0.04 | 0.03 | 0.04 | 0.03 | 0.04 | -0.02 | -0.03 | -0.10 | 0.07 | 0.09 | 1 |  |  |  |  |  |
| lwlf | -0.10 | 0.02 | -0.04 | 0.16 | 0.08 | 0.01 | -0.01 | -0.02 | 0.03 | 0.01 | 0.07 | -0.07 | 0.15 | 0.24 | 0.54 | 1 |  |  |  |  |
| mb | -0.13 | 0.13 | 0.04 | 0.21 | 0.07 | 0.06 | 0.02 | 0.03 | -0.01 | 0.02 | 0.01 | -0.04 | 0.13 | 0.23 | 0.10 | 0.13 | 1 |  |  |  |
| mm | -0.19 | 0.13 | 0.01 | 0.15 | 0.12 | 0.07 | 0.09 | 0.00 | -0.07 | 0.04 | 0.05 | 0.09 | 0.23 | 0.21 | 0.06 | 0.12 | 0.26 | 1 |  |  |
| md | -0.05 | 0.04 | -0.03 | 0.04 | 0.05 | 0.06 | 0.05 | -0.03 | 0.00 | -0.03 | -0.04 | -0.04 | 0.03 | 0.06 | 0.09 | 0.14 | 0.03 | 0.13 | 1 |  |
| lmgen | -0.16 | 0.11 | 0.00 | 0.15 | 0.13 | 0.10 | 0.05 | -0.04 | -0.03 | 0.02 | 0.03 | 0.01 | 0.14 | 0.17 | 0.10 | 0.15 | 0.32 | 0.60 | 0.62 | 1 |

Note: d=distance to Kanha national park, e= elevation, fc=forest cover within 3km of each household, dw = distance to water, g= gender(male,female), caste= caste (upper caste, other backward classes, scheduled caste, scheduled tribe), hht = total number of people in household, land = total land area, totlvs=total livestock , gr = graze inside park (c=cow, b = buffalo, g=goat), mb = night watching, md = physical structures , mm =closer watch, lmgen=any mitigation measure used for livestock-predation, ltgr = tiger, llpd = leopard, ljkl = jackal, lwlf = wolf
