# Supplementary material for: Assessing Patterns of Human-Wildlife Conflicts and Compensation around a Central Indian Protected Area
Source: PLoS One. 2012 Dec 5;7(12):e50433. doi: 10.1371/journal.pone.0050433 (PMC3515612; doi:10.1371/journal.pone.0050433)
Supplement: Table S2 — Models included in the model sets for crop and livestock loss. (DOC) [file pone.0050433.s002.doc]

**Table S2**

| Models For Crop Raiding |
| --- |
| G. d+dw+fc+e+land+g+caste+hht+ncrop+crmn+agramt+agrtit+ma+mb+mc+me+mf |
| 1. d+dw+fc+ncrop |
| 2. d+dw+fc+e+ncrop |
| 3. ma+mb+mc+me+mf+ ncrop+d+dw+fc |
| 4. land+ncrop+ma+mb+mc+me+mf+d+dw+fc |
| 5. ma+mb+mc+me+mf+d |
| 6. d+mb+mf |
| 7. d+ncrop |
| 8. d+ncrop+crmn |
| 9. d+ncrop+crmn+land |
| 10. d+crmn |
| 11. d |
| 12. d+land |
| 13. ma+mb+mc+me+mf+ncrop |
| 14. land+ncrop+mb+mf+ma+mc+me |
| 15. ma+mb+mc+me+mf |
| 16. mb+mf |
| 17. mb+mf+land |
| 18. ma+mf+ncrop |
| 19. ncrop+crmn |
| 20. land+ncrop+crmn |
| 21. land+agramt+agrtit |
| 22. mf+caste+hht |
| 23. cmgen |
| 24. d+dw+fc+e+land+mf+caste+hht+ncrop+crmn+agramt+agrtit+cmgen |
| 25. d+dw+fc+e+land+mf+caste+hht+ncrop+cmgen |
| 26. d+dw+fc+ncrop+crmn+agramt+agrtit+cmgen |
| 27. d+dw+fc+ncrop+crmn+cmgen |
| 28. d+dw+fc+ncrop+cmgen |
| 29. d+fc+ncrop+crmn+cmgen |
| 30. d+ncrop+crmn+cmgen |
|  |
| Models For Livestock Predation |
| G. d+dw+fc+e+totlvs+g+caste+hht+land+grpab+grpag+grpac+ltgr+llpd+ljkl+lwlf+mb+mm+md |
| 1. d+dw+fc+totlvs |
| 2. d+dw+fc+e+totlvs |
| 3. d+grpac+mb+md+dw+fc |
| 4. d+mb+md+grpac+hht+dw+fc |
| 5. d+grpac+dw+fc |
| 6. d + totlvs + grpac + dw + fc |
| 7. d+grpac+mb+md |
| 8. d+mb+md+grpac+hht |
| 9. d+grpac |
| 10. d+totlvs+grpac |
| 11. d+grpab+grpag+grpac |
| 12. d |
| 13. d+mb+md+totlvs |
| 14. d+totlvs |
| 15. d+mb+md+hht+totlvs |
| 16. d+land+totlvs |
| 17. mb+md+mm |
| 18. mm+md |
| 19. mb+md+grpac |
| 20. ljkl+lwlf+ltgr |
| 21. ltgr+llpd+ljkl+lwlf |
| 22. mb+md |
| 23. grpac+hht |
| 24. grpab+grpag+grpac |
| 25. mf+caste+hht |
| 26. lmgen |
| 27. d+dw+fc+grpac+lmgen |
| 28. d+dw+fc+grpac+hht+lmgen |
| 29. d+dw+fc+grpac+totlvs+lmgen |

Note: Note: d=distance to Kanha national park, g= gender(male or female), fc= forest cover within 3km of each household, e= elevation, ncrop = number of crops, crmn=average number of cropping months ,ma= fencing, mb = night watching, mc=guard animals, md = physical structures , me =lighting, mf =scare devices, mm=closer watch, cmgen=any mitigation measure used for crop-raiding, lmgen=any mitigation measure used for livestock predation, hht = total number of people in household, land = total land area, gr = graze inside park (c=cow, b = buffalo, g=goat), caste= caste (upper caste, other backward classes, scheduled caste, scheduled tribe), agramt= agriculture land area, agrtit= legal agriculture title, totlvs=total livestock , ltgr = tiger, llpd = leopard, ljkl = jackal, lwlf = wolf. G refers to global model.
