# Supplementary material for: Assessing Patterns of Human-Wildlife Conflicts and Compensation around a Central Indian Protected Area
Source: PLoS One. 2012 Dec 5;7(12):e50433. doi: 10.1371/journal.pone.0050433 (PMC3515612; doi:10.1371/journal.pone.0050433)
Supplement: Table S3 — Models included in the model set for compensation distribution. (DOC) [file pone.0050433.s003.doc]

**TableS3**

| G. d+crf+lpf+agrtit+g+caste+ncrop+rabi+crpig+crcht+crlgr+crmac+crlpjkl+lptgr+lplpd+lpwlf+crmn+totlvs+hif+hdf+lb+land |
| --- |
| 1. d+crf+lpf |
| 2. crf+lpf |
| 3. d+agrtit+crf+lpf |
| 4. crf+lpf+ncrop+crmn |
| 5. crmn+ncrop+rabi |
| 6. crf+lpf+crpig+crcht+crlgr+crmac+crlpjkl+lptgr+lpwlf+lplpd |
| 7. crmn+ncrop+totlvs |
| 8. d+ncrop+totlvs |
| 9. crpig+crcht+crlgr+crmac+crlpjkl+lptgr+lpwlf+lplpd |
| 10. crf+lpf+crpig+crcht+crlgr+lptgr+lpwlf+lplpd+crlpjkl |
| 11. crf+lpf+crpig+crcht+lptgr+lplpd |
| 12. crpig+crcht+lptgr+lplpd |
| 13. d+crpig+crcht+lptgr+lplpd |
| 14. d+crmn+ncrop+totlvs |
| 15. d+crmn+ncrop+totlvs+crf+lpf |
| 16. d+agrtit+crf+lpf+hif+hdf |
| 17. crf+lpf+hif+hdf |
| 18. crf+lpf+crmn+ncrop+hif+hdf |
| 19. crf+lpf+crpig+crcht+crlgr+crmac+crlpjkl+lptgr+lpwlf+lplpd+hif+hdf |
| 20. crpig+crcht+crlgr+crmac+crlpjkl+lptgr+lpwlf+lplpd+hif+hdf |
| 21. crf+lpf+crpig+crcht+crlgr+lptgr+lpwlf+lplpd+crlpjkl+hif+hdf |
| 22. crf+lpf+crpig+crcht+lptgr+lplpd+hif+hdf |
| 23. d+crf+lpf+hif+hdf |
| 24. d+crmn+ncrop+totlvs+crf+lpf+hif+hdf |
| 25. hif+hdf |
| 26. crf+lpf+crpig+crcht+lptgr+lplpd+lb |
| 27. crf+lpf+crpig+crcht+crlgr+crmac+crlpjkl+lptgr+lpwlf+lplpd+lb |
| 28. crf+lpf+crpig+crcht+crlgr+lptgr+lpwlf+lplpd+crlpjkl+lb |
| 29. crf+lpf+crpig+crcht+lptgr+lplpd+hif+hdf+lb |
| 30. crf+lpf+crpig+crcht+crlgr+crmac+crlpjkl+lptgr+lpwlf+lplpd+hif+hdf+lb |
| 31. crf+lpf+crpig+crcht+crlgr+lptgr+lpwlf+lplpd+crlpjkl+hif+hdf+lb |
| 32. d+crf+lpf+lb |
| 33. d+agrtit+crf+lpf+lb |
| 34. d+crmn+ncrop+totlvs+crf+lpf+lb |
| 35. crf+lpf+lb |

Note: d=distance to Kanha National Park, crf =crop-raiding reported to authorities, lpf = livestock-predation reported to authorities, hif = human injury reported to authorities, hdf = human death reported to authorities, pig = wild pig, cht = chital, tgr = tiger, lpd = leopard, lgr = langur, wlf = wolf, mac = rhesus macaque and jkl = jackal (reported both for crop raiding and predation), agrtit= legal agriculture title, ncrop = number of crops, crmn=average number of cropping months, totlvs=total livestock, caste= caste (upper caste, other backward classes, scheduled caste, scheduled tribe) , rabi=number of rabi crops grown, g =gender, and lb= household located in legal buffer. G refers to global model.
